# High-Throughput Screening Methodology to Identify Alpha-Synuclein Aggregation Inhibitors

**DOI:** 10.3390/ijms18030478

**Published:** 2017-03-02

**Authors:** Jordi Pujols, Samuel Peña-Díaz, María Conde-Giménez, Francisca Pinheiro, Susanna Navarro, Javier Sancho, Salvador Ventura

**Affiliations:** 1 Department of Biochemistry and Molecular Biology, Autonomous University of Barcelona, 08193 Bellaterra, Spain; jordi.pujolspujol@gmail.com (J.P.); samuel.pdiaz@gmail.com (S.P.-D.); chicagpinheiro@outlook.pt (F.P.); susanna.navarro.cantero@uab.cat (S.N.); 2 Department of Biochemistry and Molecular and Cell Biology, Institute for Biocomputation and Physics of Complex Systems (BIFI), University of Zaragoza, 50018 Zaragoza, Spain; maria.conde@gmail.com (M.C.-G.); jsancho@unizar.es (J.S.); 3 Institute of Biotechnology and Biomedicine, Autonomous University of Barcelona, 08193 Bellaterra, Spain

**Keywords:** high-throughput screening, α-synuclein, Parkinson disease, amyloid, protein aggregation

## Abstract

An increasing number of neurodegenerative diseases are being found to be associated with the abnormal accumulation of aggregated proteins in the brain. In Parkinson’s disease, this process involves the aggregation of alpha-synuclein (α-syn) into intraneuronal inclusions. Thus, compounds that inhibit α-syn aggregation represent a promising therapeutic strategy as disease-modifying agents for neurodegeneration. The formation of α-syn amyloid aggregates can be reproduced in vitro by incubation of the recombinant protein. However, the in vitro aggregation of α-syn is exceedingly slow and highly irreproducible, therefore precluding fast high throughput anti-aggregation drug screening. Here, we present a simple and easy-to-implement in-plate method for screening large chemical libraries in the search for α-syn aggregation modulators. It allows us to monitor aggregation kinetics with high reproducibility, while being faster and requiring lower protein amounts than conventional aggregation assays. We illustrate how the approach enables the identification of strong aggregation inhibitors in a library of more than 14,000 compounds.

## 1. Introduction

Protein misfolding and amyloid aggregation is behind a growing number of human diseases, including Parkinson’s disease (PD) [1]. PD is the second most common neurodegenerative disorder, after Alzheimer’s disease (AD) and is still incurable. PD is characterized by protein deposition in intraneuronal inclusions, the so-called Lewy bodies (LB) and Lewy neurites (LN) [2,3], whose major component is α-synuclein (α-syn) [4], a 140 amino acid presynaptic protein encoded by the *SNCA* gene and normally found in both soluble and membrane-associated fractions of the brain [5,6]. The protein α-syn is a central component in PD pathogenesis and accordingly a privileged target for therapeutic intervention. In vitro, under physiological conditions, α-syn assembles into aggregates that are structurally similar to those found in the inclusions of disease-affected brains [7,8]. The aggregation process is thought to start from soluble monomers that polymerise into ring-shaped and string-like oligomers. These small structures coalesce to form protofibrils that assemble into insoluble fibrils [9,10]. The precise nature of the toxic α-syn species is still unclear, although it is believed that specific oligomeric species play a key role in neuronal toxicity, rather than the mature aggregates [11,12]. It is thought that the population of these small oligomeric species is also associated with the spread of the disease between different structures in the brain [13,14].

There is strong interest in the discovery of small compounds that can act as chemical chaperones modulating the aggregation of α-syn [15,16,17,18,19,20]. In the absence of a defined 3D-structure to target, screening of large collections of chemically diverse compounds is a useful approach toward the discovery of novel bioactive molecules exhibiting an α-syn anti-aggregational effect. Chemical kinetics approaches would allow the quantitative detection of the effects of potential therapeutic molecules on aggregation [21]; however, the application of this type of analysis is hampered by the low reproducibility of aggregation reactions, resulting in dissimilar kinetic parameters and/or high errors even within replicates in the same aggregation assay. This is especially true for α-syn, a protein displaying a very slow aggregation reaction, usually taking several days, which is highly influenced by factors like pH, temperature, agitation or the presence of impurities [18,19,20,22,23,24,25,26,27,28,29,30,31]. The lack of reproducibility between aggregation curves is a strong limitation to identify bona fide aggregation inhibitors, since their potency becomes hidden in overlapping errors bars, especially at the beginning of the reaction, where the more toxic oligomeric species are expected to be formed. The slow aggregation kinetics of α-syn is also an important time limitation for large-scale screening, where several thousands of potential inhibitors should be tested. Due to the dependence of the reaction on the initial protein concentration, the aggregation of α-syn can be accelerated by increasing this parameter. However, this means that very large amounts of protein will be necessary for high-throughput screening assays.

The aim of the present work is to provide a detailed aggregation kinetics protocol suitable for the large-scale screening of aggregation modulators that can be used without requiring extensive previous expertise in protein aggregation and/or in the manipulation of α-syn. By ensuring a high purity of the recombinant protein and performing protein aggregation assays in 96-well plates in presence of teflon polyballs, the fibrillation reaction is boosted, requiring times and protein quantities that are compatible with high-throughput screening. After optimizing agitation and temperature, we obtained highly reproducible kinetics that allowed us to derivate accurate aggregation constants. We illustrate how the approach permitted the identification of strong inhibitors after screening a library of more than 14,000 compounds.

## 2. Results

### 2.1. Protein Expression and Purification

**Figure 1:**
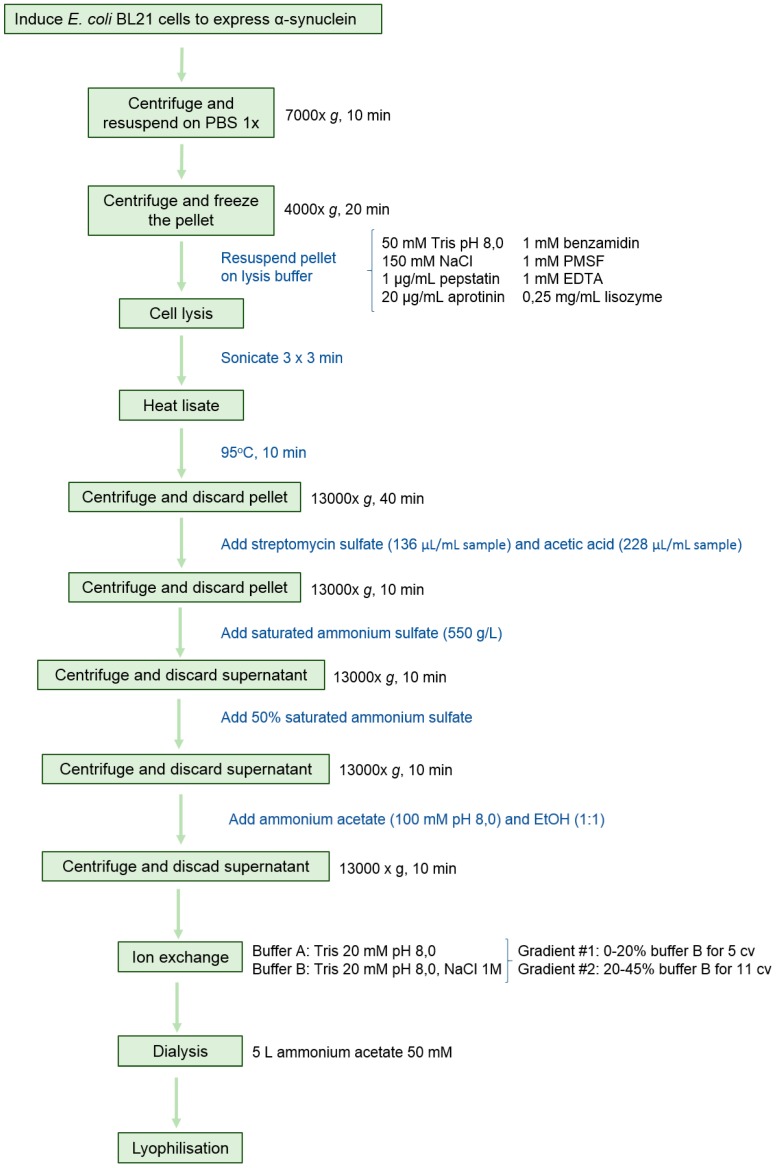
General strategy for the purification of α-synuclein.

For protein expression and purification, we adapted a protocol from Volles and Lansbury [32], including an additional sonication step during cell lysis and, more importantly, a final anion exchange chromatography (Figure 1). This purification step is crucial, since not only does it increase homogeneity, but also avoids9 the co-elution of nucleic acids. α-Syn binds to nucleic acids, the concentrations and identities of which might vary from preparation to preparation. Because most labs monitor the purity of their α-syn preparations using SDS-PAGE (Sodium Dodecyl Sulfate Polyacrylamide Gel Electrophoresis) and protein staining, nucleic acids are not visualized. The use of spectrophotometry to discard nucleic acid contaminations is highly advisable, since, in our hands, their presence results in a large heterogeneity in the kinetics of aggregation reactions.

**Figure 2:**
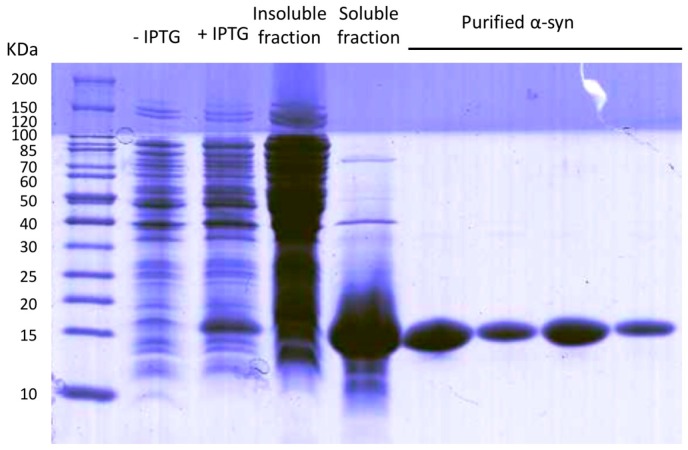
SDS-PAGE of the expression, fractionation and purification process. −IPTG and +IPTG (isopropyl β-d-1-thiogalactopyranoside) lines correspond to-non induced and induced cell extracts. The insoluble and soluble fractions of induced cells are shown as well as different fractions eluting from the anion exchange column.

Following this protocol, we obtained high amounts of nucleic acid free α-syn (35 mg/L culture). Purity of α-syn was checked by SDS-PAGE (Figure 2) and mass spectrometry, obtaining a band and a peak that corresponds to the theoretical weight of α-syn, respectively, without any trace of contaminant proteins.

### 2.2. Aggregation Kinetics

One of the most important hallmarks of α-syn aggregation is the long time it needs to be completed. When compared with other amyloid-forming proteins, the time required for the assembly of detectable mature fibrils is significantly longer, in such a way that at concentrations below 100 μM, the reaction exhibits a large lag phase, is only completed in one or more weeks and displays low reproducibility [18]. Because the aggregation ratios are extremely dependent on the initial protein concentration, the aggregation of α-syn has usually been accelerated by dramatically increasing its concentration in the assay, up to 400 μM. High concentration also reduces erratic fibrillation [9]. However, the drawback is that very large amounts of protein are necessary for large-scale screening (0.75 mg of α-syn for a single 150 μL reaction at 400 μM) [23,24].

Here we present a protocol, in which, despite the use of concentrations of purified α-syn < 100 μM, the sigmoidal aggregation reactions are completed in approx. 24 h. To track the aggregation progress, we took advantage of the amyloid specific reporter Th-T (Thioflavin-T), added to each sample at 40 μM as final concentration. One of the main problems when performing kinetics is the loss of the mixture homogeneity as the reaction progresses. Most of the assays are performed on Eppendorf or glass tubes, from where aliquots are removed at certain times and diluted for fluorescence measurements in a quartz cuvette. Dilutions do not necessarily represent the original species population, and even when samples are sonicated or re-mixed, it is impossible to predict how representative the amount of aggregate is that we are taking out from the solution. To reduce variability, caused by both sample manipulation and aggregates heterogeneity, in our case, measurements were directly recorded in a 96-well plate (black plastic) using a plate reader.

**Figure 3:**
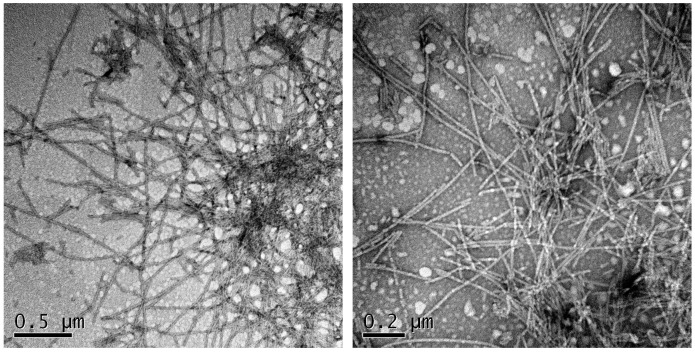
α-Synuclein fibrils formed in the presence of teflon beads. TEM images were collected upon incubation of 70 μM soluble α-syn for 24 h with agitation in the presence of beads. The samples were briefly sonicated before imaging.

Agitation has been shown to accelerate α-syn aggregation up to 1 order of magnitude. The effect of the agitation is implicitly or explicitly attributed to mass transfer or fibril fragmentation. However, for α-syn, despite agitation with only air resulting in somewhat accelerated kinetics, in our hands, the reactions were inconsistent from well to well and even resulted in different aggregates morphology. The use of glass beads during agitation potentially increases the air–water interface area; however, their effect on the speed and reproducibility of the reaction is controversial [33,34]. Investigations have demonstrated the sensitivity of aggregation reactions to hydrophobic-water interfaces and in particular to teflon-water interfaces [35] and indeed teflon beads have been used in a number of α-syn aggregation studies [36,37]. Therefore, we incorporated a teflon bead (2 mm diameter) in each well and incubated the plates under continuous agitation in an orbital shaker, since it provides more homogeneous ball agitation than a linear one. The presence of these little spheres accelerated dramatically the kinetics of α-syn aggregation, allowing us to work with 70 μM α-syn in a final sample volume of 150 μL, thus reducing the amount of protein needed per reaction (0.145 mg/well). No aggregates were observed to form at the same protein concentration and conditions during the first 24 h in the absence of mixing balls, whereas in their presence, typical long unbranched amyloid fibrils could be observed by TEM (Transmission Electron Microscopy) (Figure 3).

**Figure 4:**
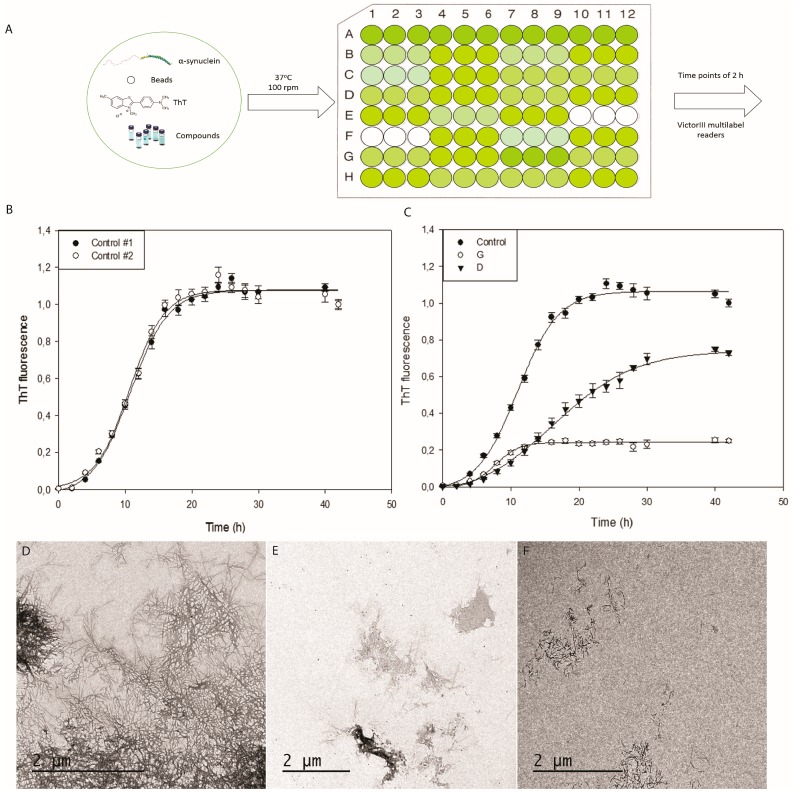
Aggregation kinetics, from plate preparation to putative inhibitors. (**A**) General scheme for plate preparation and incubation; (**B**) α-synuclein aggregation kinetics performed on non-consecutive days; (**C**) α-synuclein aggregation kinetics in presence of putative inhibitors. Measured by Th-T fluorescence emission, represented as normalised means. Error bars are represented as standard error. (**D**–**F**) TEM images of α-synuclein fibrils in absence (**D**) and presence of inhibitors (**D**–**G**).

One remarkable advantage of the protocol is the capacity to simultaneously handle a high number of plates by fixing them on a conventional orbital culture incubator, instead of using the plate reader itself to incubate the plates one by one. In this way, we can achieve a smooth, prolonged and continuous agitation, in contrast to the short and intermittent one (commonly only 15 s before each measure) performed by conventional plate readers. Moreover, it is possible to accurately adjust the temperature (37 °C) and agitation conditions (100 rpm), enabling us to acquire well-to-well and plate-to-plate reproducible aggregation curves. Plates are only pulled out from the incubator for fluorescence reads and rapidly returned to their original location. Together, the method reduces both protein concentration and aggregation times, abolishes most sample manipulation, allows for simultaneous assays and maximizes reproducibility (Figure 4A). The accuracy of the protocol can be observed in Figure 4B, where it is shown how plotting Th-T fluorescence signal as a function of time for two different aggregation reactions, recorded in non-consecutive days, results in sigmoidal curves that overlap almost perfectly. In this way, when k1 (nucleation rate constant) and k2 (growth rate constant) were calculated using the Finke-Watzky two-step model for the two curves [38] we obtained k1_1_ = 0.00640 ± 0.0013 h^−1^, k1_2_ = 0.00645 ± 0.0016 h^−1^ and k2_1_ = 0.3623 ± 0.0264 h^−1^, k2_2_ = 0.3365 ± 0.0336 h^−1^, with correlation coefficients *R* > 0.998, in both cases. As expected, the relative standard error is larger for k1 than for k2, indicating that nucleation is inherently more stochastic than elongation. This is the reason why the use of preformed amyloid seeds usually results in highly reproducible kinetics [39]; however, this precludes the identification of compounds that target the nucleation phase of amyloid formation.

The applicability of this methodology was validated by screening a large chemical library. We used the Maybridge HitFinder Collection, containing 14,400 compounds, in the search for putative inhibitors of α-syn aggregation. Each compound was tested in triplicate and each plate contained a triplicated α-syn control devoid of compound. Discarding false positives caused by Th-T fluorescence quenching during the data collection was essential. For this purpose, we recorded the absorbance spectra from 400 to 600 nm for any putative positive compound, and discarded those absorbing either at the Th-T excitation or emission wavelengths, 450 and 480 nm respectively. The successfulness of the approach is illustrated in Figure 4C, with two compounds (D and G) that exhibit medium and high effect on final Th-T fluorescence. It can be observed the low error bars obtained for all the reactions, which results in high statistical significance when assessing the inhibitory potency of the compounds at any time point. Accordingly, fitting the experimental data to the Finke-Watzky curve resulted in the large majority of cases in correlation coefficients *R* > 0.985. The ability of the method to identify bona fide inhibitors requires an orthogonal validation. In our case it was confirmed by visualizing control and compound-treated reactions end points by TEM. As an example, it can be seen in Figure 4D–F how the presence of compounds D and G results in an important decrease in both number and size of α-syn fibrils. Centrifugation assays indicated higher levels of soluble α-syn at the end of the reaction in the presence of inhibitors. The screening rendered 47 novel compounds able to efficiently modulate the aggregation and inhibit fibril formation of α-syn. These compounds belong to different chemical families, supporting the versatility of the assay. The chemical structures and the activities of 10 representative molecules are illustrated in Table 1. After fitting their respective aggregation curves, the 47 compounds fall into one of two classes: those that decrease the final Th-T fluorescence without reducing k1 and k2, like compound G, and those that decrease both the fluorescence signal and the kinetic constants, like compound D. They are those last compounds that exhibit pharmacological interest, since they are expected to delay the onset of the aggregation reaction. A convenient way to discriminate between these two types of molecules in large-screening assays is to compare the halftime of aggregation (*t*_50_) [40].

**Figure 5:**
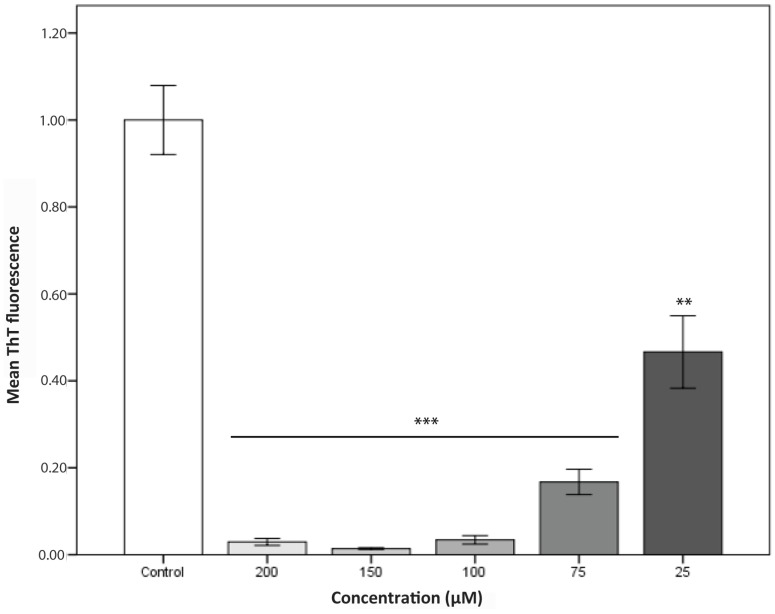
Inhibition of α-synuclein aggregation at different concentrations of the compound D. Error bars are represented as standard error, where *p* < 0.005 and *p* < 0.0005 were indicated by ** and *** respectively.

The present method not only provides an effective system for screening, but can be used to set up a variety of experiments. For instance, when developing new anti-aggregational drugs, it is essential to titrate their concentration-dependent activity. Many promising potential drugs fail because they are not effective at a concentration compatible with their pharmaceutical application. The high reproducibility of the method allows us to perform accurate titration assays, as illustrated for compound D in Figure 5 where a clear dose-dependent anti-aggregation activity could be tracked for this molecule, which turns out to be active even at sub-stoichiometric protein:compound ratios. However, we should clarify that the method only allows us to detect α-syn species that can be stained with Th-T and thus it fails to monitor the impact of the compounds in the formations of early oligomers.

## 3. Discussion

The present work provides a simple and accurate method for screening large chemical libraries in the search of lead compounds with the potential to become enrolled in future treatments for PD. It is conceived as a friendly-to-use, easy-to-implement, protocol for non-specialized research labs, with the aim of reducing both time and resources, without losing accuracy. It allows us to monitor aggregation kinetics with high reproducibility and low errors, permitting to identify true positives among large collections of putative candidates and, in addition, assessing specific drug features such as the mechanism of action or the concentration and time dependence of the compound activity. Despite it is true that compounds able to inhibit in vitro α-syn in the presence of teflon polyballs would not necessarily behave in the same manner in vivo and thus that they are still far from being biologically effective therapeutics for synucleinopathies, the possibility to recruit novel labs in the search for such molecules, without the need for an initial large investment in specialized equipment, would likely boost the finding of novel candidates with the potential to halt the onset or the progression of these devastating disorders.

## 4. Materials and Methods

### 4.1. Expression and Purification of Human α-Synuclein

Human α-synuclein was expressed and purified adapting a previous protocol from Volles and Lansbury [32]. *Escherichia coli* BL21 (DE3) cells were transformed with a pET21a plasmid (Novagen, EMD-Millipore, Darmstadt, Germany) containing the α-syn cDNA, grown in LB medium containing 100 μM/mL ampicillin and induced with 1 mM IPTG for 4 h at an optical density at 600 nm of 0.6. After cell centrifugation at 7000× *g* for 10 min at 4 °C, the pellet was resuspended in 20 mL Phosphate Buffered Saline (PBS) buffer, centrifuged again at 4000× *g* for 20 min at 4 °C and frozen at −80 °C. When needed, the pellets were defrosted and resuspended in 10 mL lysis buffer (50 mM Tris pH 8, 150 mM NaCl, 1 μg/mL pepstatin, 20 μg/mL aprotinin, 1 mM benzamidine, 1mM PMSF, 1 mM EDTA and 0.25 mg/mL lysozyme) prior to sonication using a LabSonic^®^U sonicator (B. Braun Biotech International, Melsungen, Hessen, Germany) with a power level of 40 W and a repeating duty cycle of 0.7 s for 3 intervals of 3 min. Resultant cell extract was boiled at 95 °C for 10 min and centrifuged at 20,000× *g* for 40 min at 4 °C. To the obtained supernatant 136 μL/mL of 10% *w*/*v* streptomycin sulfate and 228 μL/mL of pure acid acetic were added and centrifuged at 4 °C (20,000× *g*, 10 min). The resulting soluble fraction was diluted with saturated ammonium sulfate (550 g/L) 1:1 (*v*/*v*) and centrifuged at 4 °C (20,000× *g*, 10 min). Then, the pellet was resuspended in 50% ammonium sulfate and centrifuged at 4 °C (20,000× *g*, 10 min). The pellet was washed with 100 mM pH 8 ammonium acetate (5 mL per culture litre) and pure EtOH 1:1 (*v*/*v*), then, the mixture was centrifuged at 4 °C (20,000× *g*, 10 min). The pellet was resuspended in 20 mM pH 8 Tris and filtered with a 0.45 mm filter. Anion exchange column HiTrap Q HP was coupled to an ÄKTA purifier high performance liquid chromatography system in order to purify α-synuclein. Tris 20 mM pH 8 and Tris 20 mM pH 8, NaCl 1 M were used as buffer A and buffer B respectively. After column equilibration with buffer A, sample was injected by using a Pump Direct Loading P-960 and the weak bonded proteins were washed with 5 column volumes (cv) of Buffer A. To properly isolate α-syn, a step gradient was applied as follows: (i) 0%–20% buffer B, 5 cv; (ii) 20%–45% buffer B, 11 cv; (iii) 100% buffer B, 5 cv, obtaining pure α-syn between 25% and 35% buffer B concentration. The collected peaks were dialyzed in 5 L ammonium sulfate 50 mM overnight. α-Syn concentration was determined measuring the absorbance at 280 nm and using the extinction coefficient 5960 M^−1^·cm^−1^. Purity was checked using 15% SDS-PAGE and unstained Protein Standard markers from Thermo Fisher Scientific. The gel was stained with comassie brilliant blue. Identity was checked by mass spectrometry. 2 μL of protein were dialyzed for 30 min at room temperature using 20 mL of 50 mM ammonic bicarbonate and a 0.025 μm pore membrane (EMD-Millipore, Darmstadt, Germany). After that, MALDI-TOF was analysis was performed with a ground steel plate and 2,6-dihidroxiacetophenone acid as a matrix, in a MALDI-TOF UltrafleXtreme (Bruker Daltonics, Billerica, MA, USA). A 1:1 sample:matrix mixture was used, adding just 1 μL of these sample to the plate. For the analysis, a lineal mode was used with an accelerated voltage of 25 kV. Finally, after lyophilisation, the protein was kept at −80 °C.

### 4.2. Quenching Analysis of Compounds

The different compounds were dissolved at 50 mM in pure 100% DMSO solution. In order to check the interference of the compounds with thioflavin-T (Th-T) excitation or emission, the absorption spectra for each compound at 100 μM was measured from 400 to 600 nm in a spectrophotometer Cary100.

### 4.3. α-Synuclein Aggregation and Thioflavin-T Assays

Previously lyophilised α-synuclein was carefully dissolved in PBS buffer to a final concentration of 210 μM and filtered through a Millipore s 0.22-μm filters. α-syn aggregation assay was performed in a 96 wells plate (non-treated, black plastic) containing in each well a teflon polyball (1/8′′ diameter), 40 μM thiofalvin-T, 70 μM α-synuclein, 100 μM of the tested compounds and PBS up to a final volume of 150 μL. Plates were fixed into an orbital culture shaker Max-Q 4000 Thermo Scientific (Waltham, MA, USA) to keep the incubation at 37 °C, 100 rpm. Every 2 h, the fluorescence intensity was measured using a Victor3.0 Multilabel Reader, PerkinElmer (Waltham, MA, USA) by exciting the mixtures with 430–450 filter and collecting the emission intensity with 480–510 filter (triplicates for each measurement). Each plate contained 3 α-syn controls in the absence of any compound. The averaged Th-T fluorescence obtained for these wells at the end of the experiment was normalized to 1 and the kinetic curves in the different wells re-scaled accordingly. Re-scaled curves were used to compare the controls with the effect of compounds and to ensure that the controls were reproducible between different experiments.

For the titration assay the following concentrations for all selected compounds (200, 150, 100, 75 and 25 μM) were used.

### 4.4. Transmission Electron Microscopy (TEM)

α-Synuclein fibers from final point reaction (either in absence or presence of the final concentration inhibitors) were collected and after diluting the aggregated α-syn to a concentration of 10 μM α-synuclein, each sample was sonicated for 10 min. A volume of 5 μL of these samples were placed on carbon-coated copper grids and allowed them to stand for 5 min. Then, samples were carefully dried with filter paper to remove the excess of sample. Grids were washed twice with MiliQ water by immersion and stained by incubating grids with 5 μL 2% (*w*/*v*) uranyl acetate for 2 min for the negative staining. After removal of the uranyl acetate excess with filter paper, grids were left to air-dry for 10 min. The samples were imaged using a Transmission Electron Microscopy Jeol 1400, JEOL (Peabody, MA, USA) operating at an accelerating voltage of 120 kV. At least 30 fields were screened, to obtain representative images.

### 4.5. Statistical Analysis

Data were analysed by ANOVA Tukey test using SPSS software version 20.0 (IBM Analytics, Armonk, NY, USA). All data are shown as means and standard error. *p* < 0.05 was considered statistically significant and indicated by ** and *** if *p* < 0.005 and *p* < 0.0005, respectively.

## Figures and Tables

**Table 1:** Representative active compounds identified in the high-throughput screening.

Name	Code	Structure	% Inhibition ^a^	*t*_50_ (h) ^b^
*N*-(1-benzothiophen-2-yl)-*N*′-[3,5-bis(trifluoromethyl)phenyl]urea	A	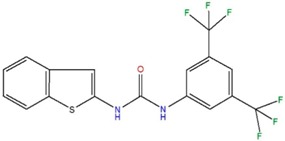	79.6	1
5-bromo-*N*-(3-chloro-4-fluorophenyl)-2-hydroxybenzamide	B	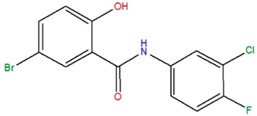	74.4	5
1-[4-(4-chlorophenyl)-2,5-dihydro-1,3-thiazol-2-yliden]-2-(1-methylethylidene)hydrazine	C	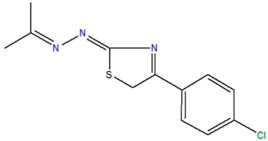	76.4	−3
2-hydroxy-5-nitro-6-(3-nitrophenyl)-4-(trifluoromethyl)nicotinonitrile	D	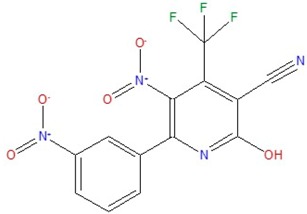	32.4	3
4-(4-methoxyphenyl)-5-(2-thienyl)-2,4-dihydro-3H-1,2,4-triazole-3-thione	E	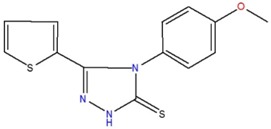	59.8	3
methyl 6-(4-methoxyphenyl)-2H-thiopyran-3-carboxylate	F	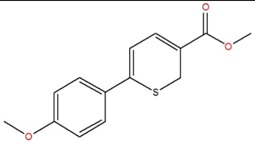	93.0	1
4-cyclohexyl-2-{[2-nitro-4-(trifluoromethyl)phenyl]thio}-6-oxo-1,6-dihydropyrimidine-5-carbonitrile	G	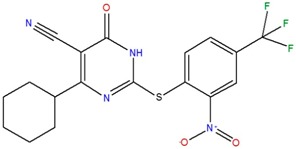	72.7	−2
3-(2-methyl-2,3-dihydro-1-benzofuran-5-yl)-5-(trifluoromethyl)-4,5-dihydro-1H-pyrazol-5-ol	H	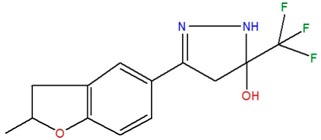	31.7	1
4-[4-(1H-pyrrol-1-yl)phenyl]-6-(trifluoromethyl)pyrimidin-2-amine	I	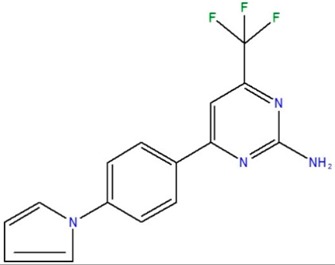	0	11
5-chloro-2-(4-nitrophenyl)benzo[b]furan	J	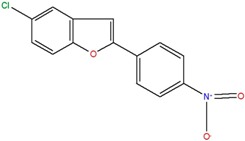	44.2	1
benzyl *N*-{[3,5-bis(methylsulfanyl)-4-isothiazolyl]carbonyl}carbamate	K	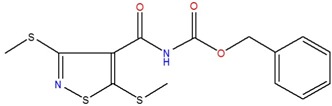	67.7	5

^a^ Measured as the change in Th-T fluorescence at the end of the aggregation reaction relative to the control; ^b^ ∆*t*_50_ corresponds to the difference between the *t*_50_ of the reaction in the presence and the absence of compound.

## Abreviations

α-syn	α-synuclein
PD	Parkinson Disease
AD	Alzheimer’s disease
Th-T	Thioflavin T
TEM	Transmission Electron Microscopy
cv	column volumes
